# China's Stem Cell Research and Knowledge Levels of Medical Practitioners and Students

**DOI:** 10.1155/2021/6667743

**Published:** 2021-05-18

**Authors:** Deng Luo, Zihui Xu, Zhongjing Wang, Wenzhuo Ran

**Affiliations:** ^1^Department of Endocrinology, Renmin Hospital of Wuhan University, Wuhan, China; ^2^Department of Endocrinology, The Central Hospital of Wuhan, Tongji Medical College, Huazhong University of Science and Technology, Wuhan, China; ^3^Department of Clinical Laboratory, Wuhan No. 1 Hospital, Tongji Medical College, Huazhong University of Science and Technology, Wuhan, China

## Abstract

Over the last few decades, China has greatly expanded its scope of stem cell research, generating various scientific advances and medical applications. However, knowledge of the extent and characteristics of domestic stem cell development, particularly medical workers' opinions, is lacking. This study's purposes were to analyze the growth trends of China's stem cell community and identify the knowledge and attitudes held by Chinese medical workers regarding stem cell research. We found that there are currently 13 high-quality stem cell research centers with more than 400 PhD-level researchers across Mainland China. These centers feature many high-caliber scientists from the stem cell research community. From 1997 through 2019, the National Natural Science Foundation of China allocated roughly $576 million to 8,050 stem cell programs at Chinese universities and research institutions. China's annual publications on stem cells increased from less than 0.6% of the world's total stem cell publications in 1999 to more than 14.1% in 2014. Our survey also revealed that most participants held positive attitudes toward stem cell research, supported further funding, and had high general awareness about stem cells.

## 1. Introduction

Stem cells are immature cells capable of becoming any cell type through the process of differentiation [1]. When injuries occur, these super cells can replicate rapidly and then mature into different cells needed around the body to repair and rebuild damaged tissues [2, 3]. Growing interest in the future medical application of stem cell technology is leading to the emergence of a new field called stem cell science. Scientists advocate that stem cells could tackle major degenerative diseases, such as arthritis, stroke, heart disease, diabetes, cancer, Alzheimer's disease, and Parkinson's disease [4–6]. In addition, it may be possible to use stem cells to treat debilitating spinal cord injuries and other structural injuries [7, 8]. Indeed, a recent clinical trial of combination therapy in patients with newly diagnosed multiple myeloma by using patients' own stem cells is a prominent and early success [9]. Besides, stem cells will also have applications in the discovering and testing of new drugs [10]. Therefore, stem cell research holds great promise in future medicine.

Because the work has the potential to revolutionize the way that human diseases are treated, many nations, including the USA, the UK, and Japan, have invested heavily in stem cell research and its applications [11–13]. China has also increased funding in the field via multiple sources since 1997 [14–16]. According to a report by the UK-China Stem Cell Partnership Development Initiative, the awards will be jointly funded by the UK Medical Research Council (MRC) and the National Natural Science Foundation of China (NSFC): “Funding is available for the research project as well as for the essential partnership activities required to support delivery of the collaborative research program.” Up to £2 million is available from the MRC, and roughly 3 million RMB is provided by the NSFC [17].

China is emerging in the stem cell science [18]. However, knowledge of the extent and characteristics of the domestic stem cell research community, particularly China's public opinion on stem cells, is lacking. In this project, we collected and reviewed historical investigation data on the development of stem cell science in China from 1997 through 2019, as well as a survey of 32 universities and institutions conducted from December 2013 through August 2019 as part of the National Social Survey Program, which is a cross-country collaboration between universities and research institutions. Exact numbers of high-quality scientists and research centers will provide more specific or detailed information to help integrate resources and promote collaboration and communication among international and local researchers. Furthermore, by analyzing the respondents' survey results, we are helping to evaluate the levels of knowledge that clinical practitioners and medical students have about the potential of stem cells.

## 2. Materials and Methods

### 2.1. Data Collection

To explore the current state of stem cell research in China, we collected publicly accessible data from university and faculty websites about the composition of well-known stem cell laboratories at leading academic institutions. Elite scientists who worked for each research center were identified from public departmental listings or their laboratory's website. The NSFC-allocated funds and the number of programs were downloaded from http://www.nsfc.gov.cn/. The number of articles published was obtained from the ISI Web of Knowledge database. The survey study was launched by Renmin Hospital of Wuhan University. A total of 32 universities representing China's geographical regions participated; a questionnaire was distributed to the selected medical colleges' practitioners and students randomly. Survey responses were anonymous and 2,310 responses were received.

### 2.2. Statistical Analysis

The mean, range, and shape of the distribution were examined for each continuous variable, with frequencies and percentages (%) tabulated for each categorical variable. All analyses were performed using SPSS Statistics 22.0.

## 3. Results

### 3.1. China's Top Stem Cell Research Centers and Currently Most Influential Scientists

**Figure 1:**
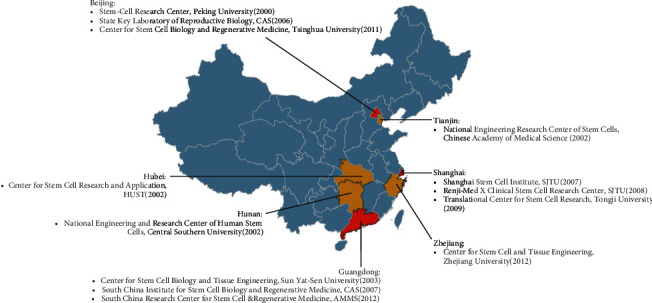
Leading stem cell research centers in mainland China. Brackets show the year of establishment.

Twenty years ago, few scientists were involved in the field of stem cells in China; today, more than 400 Chinese PhD-qualified researchers are working on a variety of stem cells, and there are 13 high-quality stem cell research centers (Figure 1), including Peking University's Stem Cell Research Center and the Institute of Zoology at the Chinese Academy of Sciences (CAS) (both in Beijing); the National Engineering and Research Center of Human Stem Cells at Changsha's Xiangya Medical College; the National Engineering Research Center of Stem Cells, which is affiliated with the Chinese Academy of Medical Sciences; Guangzhou's Center for Stem Cell Biology and Tissue Engineering at Sun Yat-Sen University; Tongji Medical College's Stem Cell Research and Application Center in the Wuhan Union Hospital at Huazhong University of Science and Technology (HUST); Renji-Med X Clinical Stem Cell Research Center at Renji Hospital, Shanghai Stem Cell Institute of Shanghai Jiao Tong University (SJTU) School of Medicine, and Tongji Hospital's Translational Center for Stem Cell Research at Tongji University School of Medicine (all in Shanghai); the South China Institute for Stem Cell Biology and Regenerative Medicine, the Guangzhou Institutes of Biomedicine and Health; the Center for Stem Cell Biology and Regenerative Medicine, which is affiliated with Tsinghua University; the South China Research Center for Stem Cell & Regenerative Medicine, which is affiliated with the Academy of Military Medical Sciences (AMMS); and Zhejiang University's Stem Cell and Tissue Engineering Center.

These China's top research centers and laboratories have a number of local stars of the Chinese stem cell research community: Hongkui Deng at Peking University, Guangxiu Lu at Xiangya Medical College, Peng Xiang at Sun Yat-Sen University, Zhongchao Han at the Chinese Academy of Medical Sciences and Peking Union Medical College, Ying Jin at the Shanghai Stem Cell Institute (affiliated with SJTU), Weiqiang Gao at Renji Hospital (also affiliated with SJTU), Qi Zhou at the Institute of Zoology, Shiang Huang at HUST, Duanqing Pei at the CAS, Qimin Zhan at the Chinese Academy of Medical Sciences and Peking Union Medical College, Yi Sun at Tongji University School of Medicine, Duanqing Pei at the Guangzhou Institutes of Biomedicine and Health (affiliated with the CAS), Wei Guo at Tsinghua University, XueTao Pei at the AMMS, and HongWei Ouyang at Zhejiang University. Fanyi Zeng at Shanghai Stem Cell Institute of SJTU is mainly engaged in developmental biology and medical genetics, with reprogramming cells and molecular mechanisms in mammalian reproduction and development as the core. Zeng's research has been published in more than 40 papers in *Nature*, *PNAS*, and other authoritative academic journals [19–21].

### 3.2. The Pattern of Funding Growth in China for Stem Cell Research

**Figure 2:**
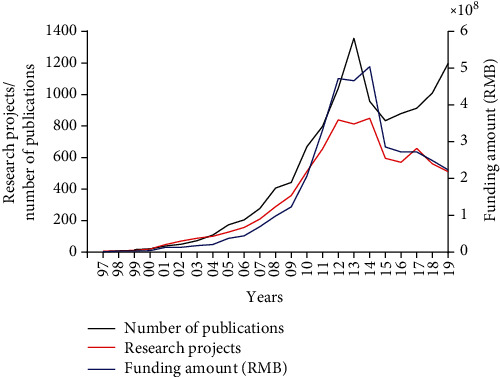
Publications and funding on stem cells in China from 1997 to 2019. Black line shows the trend of publications. Red line shows the trend of research projects. Blue line shows the trend of funding amount.

For more than a decade, China's government has devoted the majority of its science funding to stem cell research. The NSFC is a funding organization responsible for the management of the National Natural Science Fund and is aimed at promoting and financing basic research and its applications in China. To understand better the pattern of funding growth, the NSFC's data of stem cell funding from 1997 to 2019 were collected. According to the NSFC, the total amount of government spending on stem cell research from 1997 to 2019 has been about 3.7 billion yuan (roughly $576 million). Funding on a yearly basis increased dramatically, rising from 0.69 million yuan in 1997 to about 504 million yuan in 2014. Growth is slow and steady at an early developmental stage, but there is a fast growth between 2006 and 2012. Our analysis revealed that the number of yearly research programs increased over fivefold from 156 in 2006 to 839 in 2012. However, both the program numbers and funding amount were decreased after 2014 (Figure 2). This reflects that China's stem cell research has got into an adjustment period (2015-2019).

**Figure 3:**
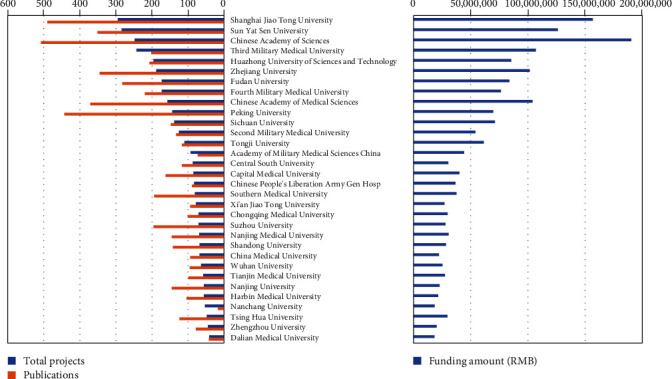
Top 32 China's universities and institutions in stem cell research, 1997–2019. Left histograms show their projects (blue) and publication (orange) on stem cells. Right histograms show their funding amount on stem cells.

China's leading universities and research institutions are taking steps to enhance their stem cell research. The NSFC allocated 295 stem cell programs to SJTU between 1997 and 2019, which were 3.7% out of the whole programs (*n* = 8,050). Besides, Sun Yat-Sen University, the CAS, and the Third Military Medical University were 284, 248, and 243, respectively. However, the CAS was allocated the most funding amount of over 190 million RMB (Figure 3).

### 3.3. Trends of Publications on Stem Cells by Chinese Scholars

From 1999 to 2014, China's annual publications on stem cells jumped from 16 to 957; this represents a growth from 0.6% to more than 14.1% of the world's total stem cell research publications. Although the number of publications has a short decline in 2014 and 2015, there is a recovery growth in the adjustment period (2015-2019) (Figure 2).

In addition, we also analyzed the publications on stem cells of China's top universities and agencies. We compared the relative contribution of various institutions in different periods. In the early period from 1997 to 2002, the Chinese University of Hong Kong and its affiliated Prince of Wales Hospital occupied the top 2 positions in stem cell publications. However, mainland Chinese universities and agencies developed rapidly and replaced top positions in the next period. The CAS topped the list, followed by SJTU and Peking University, while Chinese Academy of Medical Science, Sun Yat-Sen University, Zhejiang University, Fudan University, and the Military Medical Universities also show high levels of activity in stem cell research (Figure 3). Being overtaken by mainland universities indicated that China funding reforms had promoted a shift in research emphasis.

**Figure 4:**
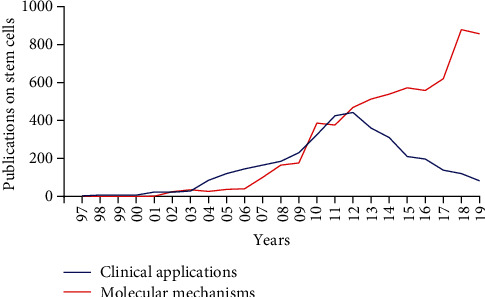
Trends of publications on stem cell clinical applications and molecular mechanisms by Chinese scholars from 1997 to 2019. Red line shows the trend of publications on molecular mechanisms. Blue line shows the trend of publications on clinical applications.

In this study, we also performed an analysis of the trends of research categories in stem cells (Figure 4), for example, the clinical applications or molecular mechanisms of stem cells. The stem cell research responsible for molecular mechanisms increased quickly from 1997 to 2019, but publications focused on clinical applications remained relatively unstable during the same period. In particular, after 2012, clinical application publications decreased gradually.

### 3.4. China's Organoids Research

**Figure 5:**
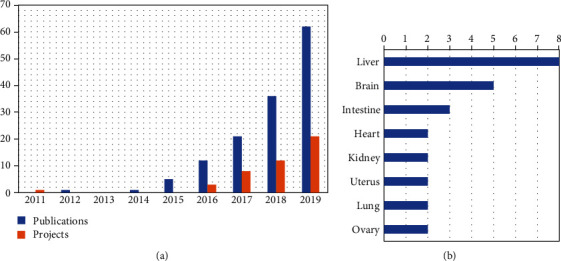
China's organoids research. (a) The trends of publications (blue) and projects (orange) on organoids research in mainland China from 2011 to 2019. (b) The organs and their frequencies involved in these organoids projects.

Organoids research is a new field of stem cell science. Organoids are self-renewing and self-organizing 3-dimensional cellular structures that resemble organs in function and structure. They can be derived from embryonic stem cells, induced pluripotent stem cells, or adult stem cells [22]. From 2011 through 2019, the NSFC has allocated 29.68 million RMB to 45 organoids projects. In general, the number of projects and publications showed a fast upward trend. In 2011, China funded the first organoids project. In 2016, 2017, 2018, and 2019, NSFC funded 3, 8, 12, and 21 organoids projects, respectively (Figure 5(a)). This increasing trend is highly consistent with the development of organoids research in the world. In these 45 projects, the 8 most studied organs are the liver (*n* = 8), followed by brain (*n* = 5), intestine (*n* = 3), heart (*n* = 2), uterus (*n* = 2), ovary (*n* = 2), kidney (*n* = 2), and lung (*n* = 2) (Figure 5(b)). The 5 most allocated universities and institutions are Zhejiang University (8.365 million RMB), Second Military Medical University (3.165 million RMB), CAS (2.41 million RMB), Chongqing Medical University (2.4 million RMB), and Fudan University (2.35 million RMB). It shows that Chinese scholars have done a lot of research on in vitro 3D culture of different types of stem cells to establish organoid models, which are mainly used for in vitro drug screening. In terms of uses, precision medicine, tumor research, and personalized medicine dominate.

### 3.5. Participants' Knowledge Levels and Opinions of Stem Cell Research

**Figure 6:**
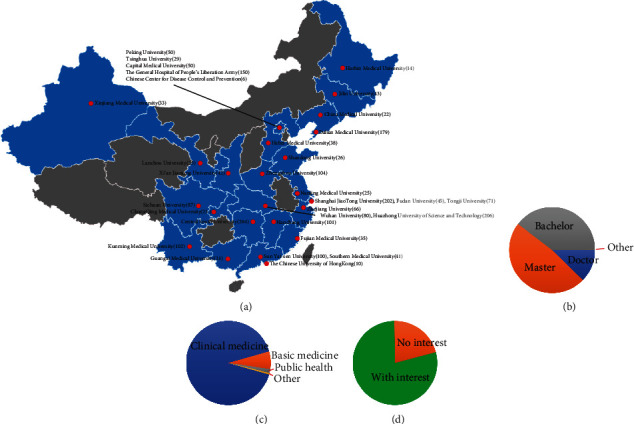
Participants' knowledge levels and opinions on stem cell research. (a) Surveyed 32 universities and institutions from 24 cities in different regions of China. Brackets show numbers of participants. (b) Educational background of participants. (c) Majors of participants. (d) Participants' interest in stem cells.

A total of 1,668 (72.2%) medical students and 642 (27.8%) clinical practitioners participated in the National Stem Cell Research Survey (Table 1). Participants were recruited from 32 universities with varying levels of academic performance in 24 cities that are representative of China's different geographical regions (Figure 6). Of the 2,310 participants, 1,183 (51.2%) were men and 2,132 (92.3%) were under 30 years of age. Most of the participants have a bachelor's degree or higher: doctoral degree (12.3%), master's degree (48.1%), and bachelor's degree (39.5%). Among the 2,310 participants, 2,105 were in clinical medicine (91.1%), 156 were in basic medicine (6.8%), 34 were in public health (1.5%), 9 were in other majors, and 6 were from unknown departments.

For all participants, 99.1% knew stem cells, and the media (Internet, newspapers, magazines, and TV/radio) were the most common ways to acquire knowledge on stem cells. A total of 78.9% had a great interest in stem cells, while only 21.1% had no interest. Only 20.6% of participants were familiar with stem cell transplantation for human diseases, but 79.4% had low or moderate self-estimated knowledge concerning stem cell transplantation. Roughly 63.5% accepted medical research using human embryonic stem cells; 30.8% considered this morally unacceptable. In addition, 54.2% supported the research of induced pluripotent stem cells upon explanation of the nature. 71.6% supported the clinical translation of stem cells, and only 1.1% of participants were opposed. Finally, it is worth mentioning that 89.9% supported increased funding for stem cell research; fewer than 2% of those questioned were against any stem cell research funding.

## 4. Discussion

Stem cell technologies are often described as scientific breakthroughs that could potentially revolutionize medicine [23]. This study's aim was to examine the levels and characteristics of stem cell research in mainland China. First, we ascertained the number of top stem cell research centers and top-tier scientists in mainland China today. Second, we collected detailed information to investigate the historical growth trend in government funding since 1997. Third, we summarized the stem cell research publications by Chinese scholars between 1997 and 2019. Then, we reviewed the organoids research in China. Finally, we obtained valuable information on the knowledge levels and opinions of China's clinical practitioners and medical students using questionnaires. In this study, we found that China's stem cell research improved significantly from 1997 to 2019. Most of the surveyed participants expressed support for stem cell research. These findings might reflect China's efforts to provide infrastructure that supports stem cell and regenerative medicine.

According to a report in 2006, the total numbers of stem cell researchers and laboratories in China were relatively small. [24] However, after 8 years of development, the number of Chinese stem cell centers had doubled in 2014, according to detailed information from public departmental listings or the laboratory's websites. We examined the top stem cell laboratories in China and found 13 high-caliber laboratories with more than 400 PhD-qualified researchers. These research centers have many competitive scientists from the stem cell research community. With the efforts by national and local governments, universities, ministries, and agencies to provide financial and research incentives, the number of high-caliber talents returning from abroad has increased dramatically. They have cooperated with international and local partners to enhance the influence of stem cell research in China.

The rapid development of China's stem cell field depends largely on the increase in governmental funding. Since 1997, China has increased funding significantly to the field of stem cell science through multiple sources. Our results found that, between 1997 and 2019, the NSFC has allocated roughly $576 million to 8,050 stem cell programs among universities and research institutions. Growth is slow and steady at the early developmental stage from 1997 through 2006, but increased rapidly from 2006. The number of annually funded programs increased over fivefold from 2006 to 2014. Additionally, the Ministry of Science and Technology of China has also provided significant research funding through National High-tech R&D Program (863 Program) and National Program on Key Basic Research Projects (973 Program). Although precise figures are hard to come by, it appears that China provided roughly $5 million to each of the 2 major programs in stem cell basic research and applications since 2002. The bulk of the spending in this field reflected China's determined efforts to advance stem cell science. While China has the scientific infrastructure to excel in this field, the importance of international collaboration for the field's development has also been underlined. In December 2013, the UK-China Stem Cell Partnership Development Initiative was launched to deliver significant 3-year research funding for internationally competitive and innovative collaborative projects between scientists from China and the UK, which allowed the pursuit of shared research interests.

China's stem cell research was generally strongly supported and can be divided into three stages. From the 1990s to 2008, China began to vigorously support stem cell research, and the supervision was relatively loose. From 2009 to 2014, the stem cell policy of China emphasized safety, and the policy orientation was based on stability. From 2015 to 2019, China's stem cell research has been further improved. Besides the fast development of basic research, the supervision of stem cell clinical research is more stringent. As new funding programs have been added over the years, competitive funding has become divided among some 100 competitive schemes overseen by about 30 different governmental departments. The Chinese government announcement noted that wastefulness and fragmented management has led to overlaps and inefficient use of funds for science and technology. On 2016, the Chinese government announced a passage of reform plans and eliminated the 863 Program and the 973 Program that fundamentally reshape research in the country.

We also evaluated the scientific production of stem cell research for the past 23 years and provided insights into the characteristics of the stem cell research publications. Data are based on the online version of the Science Citation Index (Web of Science) from 1997 to 2019. The number of stem cell papers published by Chinese scholars increased markedly between 1999 and 2014, which increased from less than 0.6% to more than 14.1% of the total publications in the world. According to an international report released by Elsevier, EuroStemCell, and Kyoto University's Institute for Integrated Cell-Material Sciences [25], the total publication volume is strikingly similar to that of the USA today. China has been the second most productive country regarding the volume of stem cell papers. Most of the papers were published mainly by Hong Kong's universities and research institutions from 1997 through 2006. However, Mainland China overtakes Hong Kong in number of published articles on stem cells between 2007 and 2014. The CAS topped the list, followed by SJTU and Peking University. This change in output suggests that the numerous government investments in education and infrastructure have improved China to the forefront of international scientific productivity.

Alongside their high general awareness of stem cells, the participants appear positive about the level of progress in stem cell research and support further funding. More than half of all respondents had a great interest in stem cell research; fewer than 2% of those questioned were against stem cell research and governmental funding. Despite China's widespread support, there are still a number of major hurdles on the stem cell research path in China. On the one hand, most of Chinese scholars are keen to publish their work in English language journals and present their findings at international conferences rather than local ones. This means that the exchange of views for promoting collaboration among local researchers is woefully inadequate, due to the lack of systematic data about the number of stem cell researchers and laboratories. On the other hand, China remains a developing country, with a per capita annual income of only $10,121 in 2019, ranking 72nd in the world. Government funding of stem cell science in China is relatively limited. Moreover, we fear that the output of stem cell papers is not always matched with the quality of Chinese research. Scientific corruption and fraud by minor scientists appear to have risen and led to additional negative publicity. Thus, there is also a need for the progressive development of appropriate legal and regulatory frameworks to allow China's stem cell research to move forward.

## 5. Conclusion

This study provided an analysis of the extent and characteristics of stem cell research development from data obtained between 1997 and 2019. The stem cell research landscape has changed considerably over these years. A critical acceleration began in 2006 when China began making rapid strides toward understanding stem cell science and the ways in which medicines can be used to treat illnesses. Furthermore, our survey of 2,310 highly educated participants also identified high-level support for stem cell research. Therefore, China could be considered a powerhouse in the international stem cell enterprise and will continue to apply research findings to clinical practice. Although we may not directly benefit from the survey's results, they will be used to help scientists, partners, policymakers, physicians, and medical students improve the awareness and resources that they have identified as being important to them.

## Figures and Tables

**Table 1:** A questionnaire on participants' knowledge levels and opinions of stem cell research.

Item	Questions
1	Have you heard of stem cells before?
2	Are you interested in research on stem cells?
3	What type of stem cells have you heard of?
4	How much do you know about stem cell transplantation?
5	What kind of attitude do you think our country should have towards the development of stem cell research in clinical treatment?
6	Do you think the country should increase funding for stem cell research?
7	Do you think it is necessary to conduct medical science popularization on stem cells among the general public?
8	Will you actively promote the application and prospects of stem cells to your patients and their families?
9	Do you support the use of stem cells derived from human embryos for basic research on clinical diseases?
10	IPS technology is a major breakthrough in the field of stem cell research. It solves difficult ethical disputes and immune rejection issues and makes stem cells a major step forward for clinical applications. How do you see its application prospects in China?
11	Have you participated in an academic conference on the application of stem cells in the field of clinical diseases?
12	Are you willing to take (or accept) continuing education courses related to stem cells?
13	Do you think stem cell clinical disease treatment has application prospects in my country?
14	What is your personal attitude towards the clinical translational treatment of clinical diseases by stem cells?
15	Do you have any concerns about the safety of stem cell transplantation to treat clinical diseases?
16	Before starting the treatment of stem cell clinical diseases, do you think it should go through a complete animal experiment demonstration?
17	If you can get a higher legal income, would you give priority to providing or recommending stem cell/regenerative treatment technologies to patients?
18	Do you agree and support the establishment of a dedicated human stem cell bank for the treatment of clinical diseases?
19	What are your relative concerns about stem cell transplantation for the treatment of clinical diseases?
20	Do you think there are any difficulties you may encounter in the actual operation of stem cell transplantation to treat clinical diseases?
21	If you have the opportunity to donate stem cells to save the lives of others, what kind of return do you expect?
22	If you are willing to donate raw materials for stem cell extraction, which of the following samples would you prefer to donate?
23	Are you willing to donate urine for basic research on clinical diseases?
24	What is your opinion on the basic research and clinical application of stem cells derived from urine?
25	Do you think that there should be corresponding remuneration for donating stem cells for clinical treatment?
26	What do you expect to maintain the effectiveness of stem cell treatment of clinical diseases?
27	If you are willing to donate stem cells, what do you want to know most?

## Data Availability

All data included in this study are available upon request by contact with the corresponding author.
